# Oxidative Stress Effects of Multiple Pollutants in an Indoor Environment on Human Bronchial Epithelial Cells

**DOI:** 10.3390/toxics11030251

**Published:** 2023-03-07

**Authors:** Yao Cheng, Dexuan Kong, Meng Ci, Yunlong Guan, Changyi Luo, Xianglan Zhang, Fuping Gao, Min Li, Gaofeng Deng

**Affiliations:** 1 State Key Laboratory of Building Safety and Environment, China Academy of Building Research, Beijing 100013, China; 2 Institute of High Energy Physics, Chinese Academy of Sciences, Beijing 100049, China; 3 China National Accreditation Insitute of Conformity Assessment, Beijing 100010, China; 4 Beijing Institute of Basic Medical Sciences, Beijing 100850, China

**Keywords:** multiple pollutants complex system, BTX, IAQ standard, cellular biology effect

## Abstract

Benzene, toluene, and xylene (denoted as BTX) are normally used in coatings, sealants, curing agents and other home decoration products, which can cause harm to human health. However, traditional studies mostly focus on the toxicity evaluation of a single pollution source, and little attention has been paid to the toxicity reports of multiple pollutants in a complex system. To evaluate the impact of indoor BTX on human health at the cellular level, the oxidative stress effect of BTX on human bronchial epithelial cells was assessed, including cell cytotoxicity, intracellular ROS, cell mitochondrial membrane potential, cell apoptosis, and CYP2E1 expression. The concentrations of BTX introduced into the human bronchial epithelial cell culture medium were determined based on both the tested distribution in 143 newly decorated rooms and the limited concentrations in the indoor air quality (denoted as IAQ) standards. Our study showed that the concentration in line with the standard limit may still pose a serious risk to health. The cellular biology effect studies of BTX showed that BTX, even at concentrations lower than the national standard limit, can still induce observable oxidative stress effects which warrant attention.

## 1. Introduction

Air pollutants contain many toxic chemicals that are always surrounding people and can cause serious diseases [1]. They exist not only in the outdoor environment, such as near motor vehicles and factories, but also in indoor environments [2]. Indoor pollution has gained increasing attention in recent years as people spend the majority of their time indoors. Pollutants can enter the body through breathing, causing harm to human health [3]. Unfortunately, coatings and plates used for interior decoration contain large amounts of organic solvents, which can lead to prolonged periods of exposure to pollution [4,5,6]. The benzene series of pollutants such as benzene, toluene, and xylene (denoted as BTX), are toxic and particularly harmful to human health [7]. They are neurotoxic and can cause many symptoms including neurasthenia, headache, insomnia, vertigo, and fatigue in the lower limbs. Furthermore, they are genotoxic and can damage DNA. Long-term exposure to BTX can lead to anemia and leukemia [8]. Hence, many countries have strict regulations regarding the concentrations of BTX and other volatile organic compounds (VOCs) in indoor environments. Various indoor environmental standards have been established to limit the concentration of pollutants, including BTX. For example, the WHO Air Quality Guidelines in Europe, the Hong Kong guidance note for IAQ (indoor air quality) management in offices and public places, and the Chinese National Standards GB 50325-2020 [9] and GB/T 18883-2022 [10] have all set limits on the concentration of BTX in indoor environments, as listed in Table 1.

The assessment of health risk is reliant on the prediction of exposure concentrations of total indoor semivolatile organic compounds (SVOCs) to occupants [11]. It is not feasible to directly assess pollutant concentration from a single source indoors. Currently, formaldehyde and VOCs, mainly from building materials, are the main sources of indoor air pollutants. The presence of VOCs such as BTX in indoor air has received less attention, though benzene is more hazardous than toluene and xylene due to its longer lasting and easily adsorbed properties. Toluene and xylene are currently used instead of benzene, as solvents or diluents for paints, coatings, and adhesives, as well as a waterproof material, due to their lower toxicity. Low-dose benzene exposure can induce genotoxicity and hematotoxicity, causing infertility, deformity, and intellectual disability [12,13]. It has also been reported that although the hazards of toluene and xylene are lower than that of benzene, sick building syndrome symptoms such as tearing, itchy eyes, dry eyes, and coughing can still occur in residential places, with reports indicating that women are more sensitive to BTX exposure than men [14]. The concentration limits of BTX have been established according to indoor air quality standards, but even concentrations lower than the national standard limit can cause harm to the human body. Hence, it is of crucial significance to assess the cellular level impact of indoor BTX on human health to determine acceptable concentration limits.

In this work, 143 BTX tests were conducted in redecorated bedrooms over three months to analyze the concentrations of BTX using gas chromatography. The distributions of each VOC were calculated to determine the extent of harm of BTX on human health in these rooms. Based on the distribution and the IAQ standards, three different concentrations of each BTX were set for the cytotoxicity study on human bronchial epithelial (BEAS-2B) cells. Studies on cytotoxicity, intracellular reactive oxygen species, mitochondrial membrane potential, cell apoptosis effect, and inflammatory cytokines were conducted to confirm the effect of BTX on BEAS-2B cells. The results can be helpful for the compilation of IAQ standards and for the improvement of human health.

## 2. Experimental Section

### 2.1. Detection Scheme

The detections of benzene, toluene, and xylene were conducted in 143 redecorated residential bedrooms within a period of 1 to 3 months, which were vacant during the renovation. The detection scope covered 7 different communities in the urban area of a certain city. The redecoration included painting the walls and replacing old furniture. The bedrooms had wooden or ceramic tile floors and aluminum alloy window frames that were well sealed. The walls were repainted with latex paint which was used in the original decoration, and the furniture was uniform, with the same brand and configuration (beds, mattresses, writing desks, chairs, etc.). The enclosed nature of the bedroom space was taken into account in the fixation test to consider the impact on pollutant concentration due to the permeation rate. The bedroom decoration materials involved in this study were commonly used materials in residential buildings, which were representative of the analysis of the impact of decoration materials on the indoor air environment.

Sampling took place from September 2018 to October 2018, with an outdoor temperature of 25–30 °C and an average indoor temperature of 28 °C. Due to the room size being less than 50 m^2^, only one gas sampling point was set up in the middle of the bedroom. Organic matter in the indoor environment was extracted using a gas sampling pump and adsorbed in a Tenax-TA (TQ-2000) tube. The sampling point was no less than 0.5 m away from the wall, and the sampling pump was positioned between 1.0 and 1.5 m away from the floor. The doors and windows were closed for at least 12 h before sampling and the temperature, humidity, and atmospheric pressure were measured with calibrated instruments. Samples were analyzed using thermal desorption gas chromatography within 14 days of collection, and this method is in compliance with GB/T 18883-2020.

### 2.2. Cell Culture and BTX Concentration

The human bronchial epithelial (BEAS-2B) cells were cultured with high glucose Dulbecco’s modified Eagle medium (DMEM, high glucose, with L-glutamine and Sodium Pyruvate) and 10% fetal bovine serum (FBS) at 37 °C and under a 5% CO_2_ atmosphere. BTX was dissolved in dimethylsulfoxide (DMSO) and further diluted with a culture medium (0.05%, *v*/*v*) to the required concentration. The final BTX concentrations in the cell culture medium are listed in Table 2.

### 2.3. Cytotoxity Study of BTX on BEAS-2B Cell

According to the test protocol, the BEAS-2B cells were inoculated into 96-well plates at 1 × 10^4^ cells per well. Different concentrations of BTX were added to the culture medium and incubated with the cells for 1, 6, 12, 24, 48, and 72 h, respectively. The cell activity was then tested with a Cell Counting Kit-8 (CCK-8).

### 2.4. Detection of Intracellular Reactive Oxygen Species (ROS) after BTX Incubation

After 24 h of incubation with different BTX concentrations, the cells were digested with trypsin without EDTA, and then pelleted via centrifugation at 1000 rpm for 5 min. Supernatant was carefully aspirated, and 1 mL of 4 °C pre-cooled PBS was added to resuspend the cells. After another centrifugation to pellet the cells, the culture medium was removed and diluted by 2′,7′-Dichlorofluorescin diacetate (DCFH-DA) with serum-free culture medium was added to a final concentration of 10 μmol/L. After 20 min of incubation at 37 °C, the cells were washed three times with serum-free culture medium, collected by centrifugation, and analyzed for fluorescence with a fluorescence microplate at 488 nm excitation and 525 nm emission wavelengths.

### 2.5. The Effect of BTX on the Mitochondrial Membrane Potential of BEAS-2B Cell

After 24 h of incubation with different BTX concentrations, cells were digested with trypsin without EDTA, and pelleted by centrifuging at 1000 rpm for 5 min. After carefully aspirating the supernatant, 1 mL of 4 °C pre-cooled PBS was added to resuspend the cells, followed by another centrifugation to pellet the cells. A total of 50 µL JC-1 was diluted with 8 mL of ultrapure water (200×), and then vortexed thoroughly. A total of 2 mL of JC-1 staining buffer (5×) was mixed with serum-free medium to form the JC-1 staining working solution. A cell suspension was made by adding an equal volume of JC-1 staining working solution and inverting it several times to mix thoroughly. The suspension was incubated at 37 °C for 20 min in a cell incubator, and then centrifuged at 600× *g* and 4 °C for 3–4 min to collect cells. The collected cells were washed twice with JC-1 staining buffer (1×).

The cells were resuspended in JC-1 staining buffer (1×) and their fluorescence was detected with a fluorescence microplate reader.

### 2.6. Cell Apoptosis Effect of BTX on BEAS-2B Cell

After incubating the cells with different concentrations of BTX for 24 h, the cell culture solution was collected into a centrifuge tube. The cells were then digested with trypsin without EDTA, and then the cells were pelleted via centrifugation at 1000 rpm for 5 min. After carefully aspirating the supernatant, 1 mL of 4 °C pre-cooled phosphate-buffered saline (PBS) solution was added to resuspend the cells, followed by centrifuging again to pellet the cells. The cells were resuspended and diluted with the PBS buffer to 1–5 × 10^6^ cells/mL. A total of 100 µL of the cell suspension was taken out and put into a 5 mL flow tube, and then 5 µL of Annexin V/FITC reagent was added and mixed well. The cells were incubated for 5 min in the dark at room temperature. A total of 10 µL of 20 µg/mL propidium iodide solution (PI) and 400 µL of PBS was added and mixed well. The fluorescence of the cells was immediately analyzed using flow cytometry.

### 2.7. Detection of Inflammatory Cytokines in BEAS-2B Cells Treated with BTX

The cells were treated with culture medium containing varying concentrations of BTX for 24 h. The cell culture supernatant was collected to measure the amount of TNF-α (Tumor Necrosis Factor α), IL-8 (Interleukin-8), and IL-6 (Interleukin-6) using ELISA. The experiments were carried out according to the instructions of the kit.

### 2.8. Detection of CytochromeP4502E1 (CYP2E1) after BTX Incubation

Reverse transcriptase-polymerase chain reaction analysis was performed on isolated RNA samples to determine relative amounts of secreted CYP2E1 between groups. Total RNA was isolated in TRIzol (TSINGKE), and total RNA was prepared via chloroform extraction and isopropanol precipitation according to the manufacturer’s recommendations. Reverse transcription was performed to transcribe and amplify mRNA into cDNA using a Reverse Transcriptase Kit (Vazyme, Nanjing, China). cDNA was used as a template in quantitative PCR with SYBR Green (Vazyme) to determine specific gene expression. Quantitative real-time PCR was performed using an SYBR Green PCR mixture and conducted with the FTC-3000. The primer sequence was as follows: CYP2E1 (forward, GACTGCCTGCTCGTGGAAATGG; reverse, GAGTTGTGCTGGTGGTCTCTGTC).

Cells were lysed with lysis buffer followed by sonication. Protein samples were separated on a 12% SDS-polyacrylamide gel, and transferred to 0.45 μm polyvinylidene fluoride blotting membranes. The membranes were blocked with 5% skim milk for 1 h and then incubated with a human CYP2E1-specific antibody (1:1000, affinity, DF6883) overnight at 4 °C. The membrane was subsequently incubated with a HRP-conjugated secondary antibody at 1:5000 for 2 h at room temperature and developed using an enhanced chemiluminescence substrate (Vayme). Visualization and imaging of the blots were performed with a FluorochemQ System.

## 3. Result and Discussion

### Test Results of BTX Concentration

**Figure 1:**
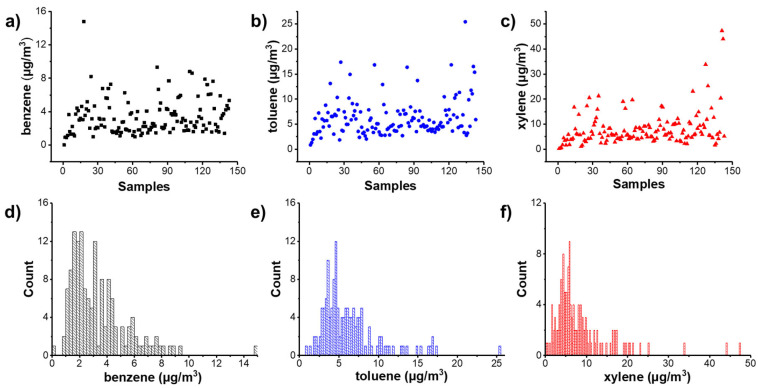
Concentration distributions of (**a**,**d**) benzene, (**b**,**e**) toluene, and (**c**,**f**) xylene in 143 indoor air samples.

The BTX concentrations were measured using the gas chromatograph method and are displayed in Figure 1. As we can see, the concentrations of BTX were all relatively low. After statistics treatments, the distribution of concentrations can be seen in Figure 1d–f. Benzene concentrations were mainly in the range of 1 μg/m^3^ to 3 μg/m^3^. For toluene, they were 0 to 7 μg/m^3^, while for xylene, they were 0 to 10 μg/m^3^. The highest concentrations of BTX were 15 μg/m^3^, 25 μg/m^3^ and 47 μg/m^3^, respectively.

**Figure 2:**
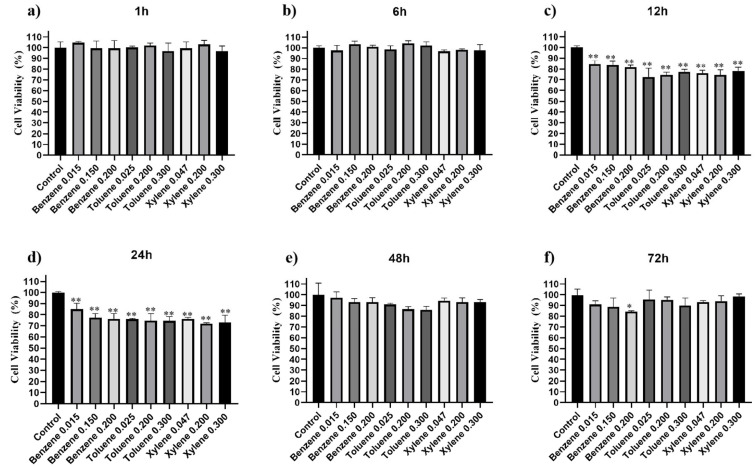
Cell viability test under different concentrations of BTX (see detailed concentrations in Table 2) and different incubation times. All BTX was dissolved in DMSO before added into BEAS-2B cell culture medium. The ratio of DMSO to cell culture medium is 0.05%. * *p* < 0.05 and ** *p* < 0.01.

The results of the cell viability experiments are shown in Figure 2. In a short period of 1–6 h (Figure 2a,b), BTX with different concentrations did not have a significant impact on cell activity. Cell viability decreased significantly when the incubation time reached 12–24 h, as shown in Figure 2c,d. However, with further extension of the incubation time, to 48–72 h, cell viability was observed to return close to the control group, as indicated in Figure 2e,f. The subtle influence of BTX on cell viability in the first few hours suggests that BTX does not severely interfere with cell proliferation, but cells still require energy to resist interference from BTX and to metabolize it. As incubation time increased, cell proliferation was pronouncedly affected by BTX, resulting in largely decreased cell viability between 12 and 24 h. Over time, the recovery of cell viability was observed, which could be due to the decreased BTX in the culture medium owing to their natural volatilization, the detoxification metabolic capacity, and the self-repair and proliferation of cells, as seen in Figure 2e,f.

It should be noted that the presence of FBS might influence the phenotype and sensitivity to toxicants in BEAS-2B, as reported by Klimecki in an arsenic toxicity study [15]. However, it is difficult to culture BEAS-2B without FBS, based on the experimental results. Therefore, the BEAS-2B cell was cultured with both high glucose Dulbecco’s modified Eagle medium (DMEM) and 10% fetal bovine serum (FBS). Though low level of FBS might still have an impact on cells, the cell viability text showed a remarkable tendency of cytotoxicity upon exposure to BTX with certain concentrations, providing information about our focus.

The above results showed that BTX had the most pronounced effect on the viability of BEAS-2B cells after 24 h of incubation (Figure 2d), suggesting that 24 h could be considered as an appropriate incubation time for further studies to investigate the impacts of BTX on cells.

**Figure 3:**
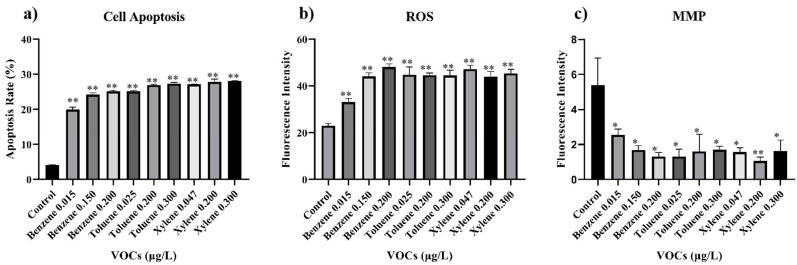
The oxidative stress effects of BTX on BEAS-2B cells were tested at different concentrations by examining (**a**) cell apoptosis, (**b**) reactive oxygen species (ROS), and (**c**) mitochondrial membrane potential (MMP) after 24 h of incubation. These factors served as indicators of oxidative stress effects. The ratio of DMSO to cell culture medium is 0.05%. * *p* < 0.05 and ** *p* < 0.01.

Figure 3a shows that BTX can significantly induce apoptosis, and the apoptosis rate increases with the increasing BTX concentration. Among the components of BTX, benzene presents the most obvious toxicity at the standard limit of 0.03 μg/L, as determined by GB/T 18883-2020, compared to toluene and xylene. The results of the apoptosis experiment are consistent with the cytotoxicity study, showing that the effects of BTX on cells ultimately lead to decreased cell viability and cell apoptosis. It is noteworthy that BTX still has significant biotoxicity on BEAS-2B cells with continuous BTX exposure at concentrations lower than the standard limit determined by GB/T 18883, as shown in Figure 2.

Figure 3b shows that BTX can also significantly increase the production of reactive oxygen species (ROS) in cells. ROS levels in BEAS-2B cells were detected using the DCFH-DA fluorescent dye, and it was revealed that benzene with a lower concentration has a similar ability to induce the production of ROS to toluene and xylene at higher concentrations. ROS can negatively impact the normal physiological activities of cells in various ways, such as damaging DNA, and oxidizing or inactivating various enzymes within the cell. ROS has a crucial role in aging and related diseases. A portion of ROS can attack the phospholipid molecules on biological membranes and proteins, damaging the structure of biological membranes or disrupting the normal function of proteins [16]. In addition to the normal cellular metabolism that generates ROS, the metabolism of various pollutants in the body contributes significantly to the increase in ROS [17]. If not repaired, DNA damage can possibly be passed on to progeny cells. The accumulation of DNA mutations during cell proliferation mainly accounts for aging or carcinogenesis [18]. The cells would be kept at a high level of ROS upon long-term exposure to a specific concentration of BTX, which results in the maintenance of high levels of ROS in cells.

The mitochondrial membrane potential is an intermediate form of energy storage produced by the conversion of redox metabolism in mitochondria. Maintaining its steady state is necessary for normal triphosadenine (ATP) production by cells. Decreased mitochondrial membrane potential can induce cell apoptosis. Figure 3c shows that exposure to BTX at different concentrations leads to a pronounced decrease in the mitochondrial membrane potential (MMP), consistent with the increased production of ROS and cell apoptosis. Despite BTX concentrations in many different places being significantly lower than the national standard limits, it still has pronounced biotoxicity on the cells.

**Figure 4:**
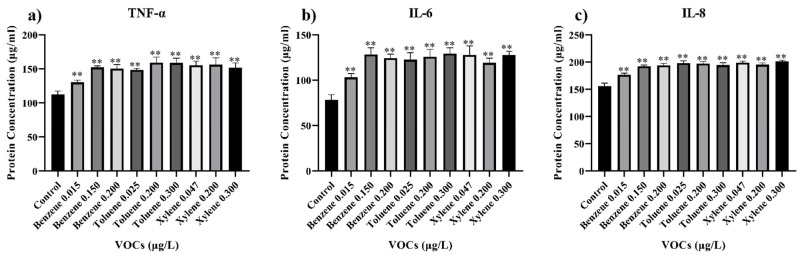
The ELISA test was used to detect the concentrations of (**a**) tumor necrosis factor-α (TNF-α), (**b**) interleukin-6 (IL-6), and (**c**) interleukin-8 (IL-8) in the supernatant of BEAS-2B cells cultured with varying concentrations of BTX. The data were collected after incubating the cells with different concentrations of BTX in the culture medium for 24 h. The ratio of DMSO to cell culture medium is 0.05%. ** *p* < 0.01.

Inflammatory factors are released cytokines after injury, which can stimulate the immune system and initiate an inflammatory response. IL-6 is a cytokine involved in inducing acute inflammation during the inflammation process. IL-8, a chemokine, helps recruit white blood cells to the site of inflammation [19]. TNF-α, which plays a role in systemic inflammation, is also a cytokine that stimulates acute inflammation [20]. Figure 4 demonstrated that even at concentration below the standard, BTX can significantly increase the release of inflammatory factors from BEAS-2B cells, indicating its toxicity. In some instances, excessive inflammation can cause more harm to the body than that caused by the disease/injury itself, such as allergic reactions and rheumatoid arthritis [21].

**Figure 5:**
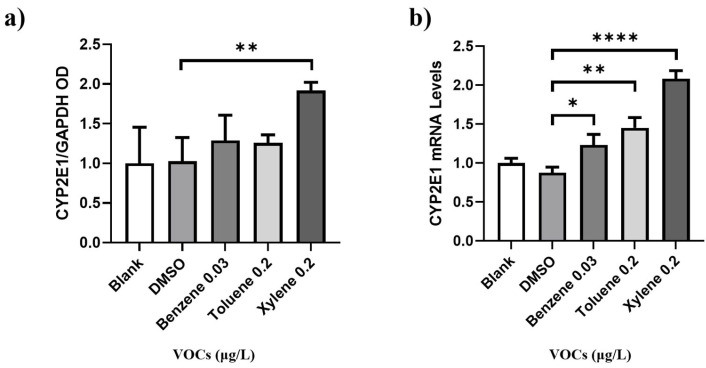
(**a**) Western blots showing CYP2E1 and GAPDH in BEAS-2B cells, which were treated with indicated doses of DMSO, benzene (0.03 μg/mL), toluene (0.2 μg/mL), and xylene (0.2 μg/mL), for 24 h. (**b**) qRT-PCR analysis of CYP2E1 in BEAS-2B cells after doses of DMSO, benzene (0.03 μg/mL), toluene (0.2 μg/mL), and xylene (0.2 μg/mL) for 24 h. Data shown are means ± SD of two independent experiments. * *p* < 0.05, ** *p* < 0.01 and **** *p* < 0.0001.

Glyceraldehyde-3-phosphate dehydrogenase (GAPDH), as a kind of house-keeping gene, is one of the enzymes in the glycolysis reaction, and shows a high expression level in almost all tissues. The expression of certain proteins in the same type of cells or tissues is usually constant and not affected by the certain recognition sites contained in it. Therefore, GAPDH is commonly used as an internal reference in quantitative RT-PCR, Western Blot, and other experiments [22]. Previous studies have shown that the toxicity of BTX is due to the formation of highly reactive metabolites that can form adducts and ROS as byproducts. Among other metabolic enzymes, cytochrome P450 2E1 (CYP2E1) is known to play a key role in BTX metabolism [23,24]. To investigate this further, we treated BEAS-2B cells with a limited concentration of BTX, under the current standard, for 24 h and analyzed the expression of CYP2E1 at both the transcriptional and protein levels. The results, as shown in Figure 5, indicate that the expression of CYP2E1 in BEAS-2B cells increased in response to BTX treatment, and with an especially significant increase for xylene treatment, confirming the toxicity of BTX on BEAS-2B cells.

### 4. Conclusions

The concentrations of BTX in 143 newly decorated rooms were measured and analyzed statistically. Benzene concentrations were primarily distributed in the range of 1 μg/m^3^ to 3 μg/m^3^, while toluene and xylene were mostly distributed in the range of 0 to 7 μg/m^3^ and 0 to 10 μg/m^3^, respectively. The highest recorded concentrations of BTX were 15 μg/m^3^, 25 μg/m^3^ and 47 μg/m^3^. Based on the test results of 143 decorated rooms and the IAQ standards, the concentrations of BTX in the BEAS-2B cell culture medium were determined. The impact of indoor BTX on human health at the cellular level was evaluated by assessing oxidative stress. Studies on cell cytotoxicity, intracellular ROS, cell mitochondrial membrane potential, cell apoptosis, and CYP2E1 expression revealed that even BTX levels below the national standard can cause observable oxidative stress effects, which may harm human health. These studies highlight the importance of considering the cumulative concentration of multiple pollutants. Even though the content of one or more types of pollutants is below the current standard, the toxicity caused by the combination of multiple pollutants in a complex system should still raise concerns. Thus, it is increasingly important to consider the cumulative concentration of multiple pollutants. These cellular biology studies provide a scientific basis for determining BTX limits in indoor environments and can aid in the development of IAQ standards.

## Figures and Tables

**Table 1:** Concentration limits of BTX in indoor air according to different standards.

Standards	Benzene	Toluene	Xylene	Ref.
WHO Air Quality Guidelines for Europe Second Edition	A safe level for airborn benzene cannot be determined. As low as possible. The concentrations of airborne benzene associated with an excess lifetime risk of 1/10,000, 1/100,000, and 1/1,000,000 are 17, 1.7, and 0.17 μg/m^3^, respectively.	The peak concentrations of toluene in air should be kept below 1000 μg/m^3^ as a 30 min average to avoid detection by odor.	-	Air quality guidelines for Europe, 2nd ed WHO
Hong Kong guidance note for IAQ management in offices and public places	For Good Class 16.1 µg/m^3^	For Good Class 1092 µg/m^3^	For Good Class 1447 µg/m^3^	A guide on indoor air quality certification scheme for offices and public places Hong Kong Environmental Protection Department
GB 50325-2020	Type-I civil building: ≤60 μg/m^3^; Type-II civil building: ≤90 μg/m^3^.	Type-I civil building: ≤150 μg/m^3^; Type-II civil building: ≤200 μg/m^3^.	Type-I civil building: ≤200 μg/m^3^; Type-II civil building: ≤200 μg/m^3^.	Indoor air quality management group of China, GB 50325-2020
GB/T 18883-2020 (exposure draft)	30 μg/m^3^ (1 h average)	200 μg/m^3^ (1 h average)	200 μg/m^3^ (1 h average)	Indoor air quality management group of China, GB/T 18883-2020

**Table 2:** BTX concentrations used in cell culture media on cell viability.

Reagents	Benzene	Toluene	Xylene
Reagent concentration (μg/L)	0.015	0.025	0.047
	0.030	0.200	0.200
	0.045	0.300	0.300

## Data Availability

The data supporting the findings of this study are available from the corresponding authors upon reasonable request.
